# Severe Vancomycin Intoxication in an Infant Not Needing Dialysis: A Case Report and Literature Review

**DOI:** 10.7759/cureus.31950

**Published:** 2022-11-27

**Authors:** Eman A Nooreddeen, Razan M Alzahrani, Nada M Alshanqiti

**Affiliations:** 1 Pediatrics, Prince Mohammed Bin Abdulaziz Hospital, Ministry of National Guard Health Affairs, Al Madinah, SAU; 2 Pediatrics, King Abdullah International Medical Research Center, Ministry of National Guard Health Affairs, Riyadh, SAU; 3 Pediatrics, King Saud Bin Abdulaziz University for Health Sciences, Riyadh, SAU; 4 Pediatrics, Ibn Sina National College for Medical Studies, Jeddah, SAU

**Keywords:** ototoxicity, kidney injury, intoxication, toxicity, vancomycin

## Abstract

Vancomycin nephrotoxicity is a major clinical concern. We report the case of an infant with severe vancomycin intoxication. A literature review was conducted due to the paucity of reported pediatric cases. An infant was treated for suspected meningitis based on cerebrospinal fluid (CSF) cell count and was empirically started on intravenous ceftriaxone and vancomycin while awaiting the results of culture and meningitis/encephalitis polymerase chain reaction (PCR) tests. Day 2 vancomycin trough level was within the target range; however, the repeat day 4 levels were beyond the upper limit of measurement at >400 µg/mL and associated with acute kidney injury (AKI). Vancomycin was immediately discontinued. The child was treated with intravenous hydration and furosemide and did not require dialysis. The short-term kidney function outcome was reassuring. We identified 23 pediatric cases from 1992 to 2021 with high vancomycin serum levels. Vancomycin level ranges between 32-427 µg/mL. Toxic vancomycin serum levels >400 µg/mL were reported in only two patients. Nephrotoxicity developed in 73.9% of cases. Hemodialysis is the most common management intervention while some patients received watchful management. Kidney function impairment is transient in most reported cases, even in those who received no intervention. However, long-term data are lacking. An intervention is not indicated for all cases of vancomycin intoxication, regardless of serum level. However, in cases of severe nephrotoxicity resistant to medical measures or pre-existing kidney dysfunction, kidney replacement therapy (KRT) is needed to manage severe AKI and speed-up vancomycin clearance.

## Introduction

Vancomycin is a glycopeptide antibiotic almost entirely excreted in urine [1]. Vancomycin exhibits variable protein binding with a median unbound fraction of 81.3% [2]. The volume of distribution is ~0.4-1.0 L/kg in adults and 0.57-0.69 L/kg in neonates [1,3]. Vancomycin clearance falls between 0.05 and 0.38 L/h/kg in critically ill children and largely depends on kidney function [4].

Measuring the minimum inhibitory concentration and targeting the “therapeutic range” of either the serum trough levels or the area under the curve can enhance efficacy and minimize the potential toxicity [5].

The incidence of vancomycin nephrotoxicity was 12.2% in a large pediatric cohort, and 19.9% if therapy lasted >3 days [6]. The risk of acute kidney injury (AKI) was linked to specific therapeutic vancomycin trough levels [7]. However, other studies did not find this association [6]. Additional risk factors for AKI in vancomycin-treated patients were reported [7].

We report the case of an infant with severe toxic vancomycin serum level >400 μg/mL who developed acute kidney injury (AKI). We defined toxic vancomycin level (TVL) as serum trough vancomycin level >30 μg/mL. The paucity of reported TVL in pediatric patients prompted us to conduct a literature review.

## Case presentation

A four-month-old girl presented to the emergency room (ER) of our center for fever. Oral cefprozil was prescribed. She was brought back the following day. She had no clear focus of infection and her C-reactive protein level was 113 mg/L. A full septic workup was performed, including a lumbar puncture. The patient did not show clinical signs of dehydration. Complete blood count results showed a white blood cell count (WBC) of 19.3 x 10^9/L, a neutrophil count of 7.14 x 10^9/L, and a lymphocyte count of 10.6 x 10^9/L. The patient was started empirically on a meningitis dose of intravenous ceftriaxone. Vancomycin 15 mg/kg/dose intravenously every six hours was added (5 mg/ml, infused over 120 min). Cerebrospinal fluid (CSF) analysis demonstrated WBC 22×106/L with 20% segmented cells, 54% lymphocytes, and 26% monocytes. CSF protein and glucose levels were normal, and the gram stain was negative. A decision was made to continue on antibiotics until the final CSF culture and meningitis/encephalitis polymerase chain reaction (PCR) results are obtained due to suspicion of partially treated bacterial meningitis. Respiratory viral PCR multiplex (RVM) results were negative. Initial urine analysis and microscopy showed specific gravity 1.005, WBC 3-5 /hpf, red blood cells 3-5 /hpf, negative nitrite and ketone, and protein trace. 

**Figure 1:**
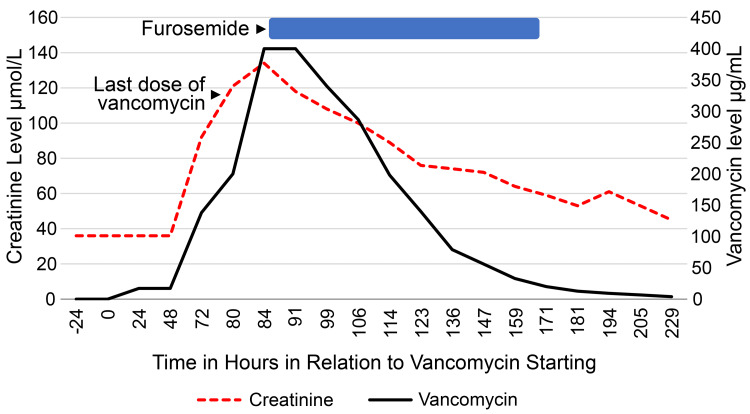
Serum levels of vancomycin and creatinine of the patient in relation to intervention

The initial serum creatinine level was 36 µmol/L. The vancomycin trough level was within the target (17.1 μg/mL) prior to the fourth dose. On day 2 of therapy, the patient developed irritability and skin reaction. Redman syndrome was suspected. Dexamethasone and diphenhydramine were administered. Vancomycin infusion time was increased to 150 min. Her symptoms did not recur. The final CSF culture and meningitis/encephalitis PCR results were still pending. On day 3, she vomited a few times, after which she could tolerate oral nutrition. She also had a fever and was administered paracetamol. She was fairly hydrated and underwent repeat vancomycin trough level and kidney function tests on day 4 for routine monitoring. The vancomycin serum level was >138 μg/mL, which was beyond the upper limit of measurement (BULM) and serum creatinine had increased to 92 µmol/L; vancomycin was discontinued. On the same day, the final CSF culture and meningitis/encephalitis PCR results were negative. She was given one intravenous isotonic saline bolus of 10 ml/kg and then admitted to the pediatric intensive care unit for close monitoring and management. During the furosemide and fluid therapy, repeat vancomycin random serum levels continued to be BULM (>400 μg/mL after sample dilution) and serum creatinine was rising (Figure 1). The kidneys were enlarged on ultrasonography but with preserved cortico-medullary differentiation. Repeat RVM results were positive for rhinovirus which explains the development of transient fever during day 3 of admission. 

The parent was counseled regarding hemodialysis (HD) but refused. Another two intravenous isotonic saline boluses were administered, one as 10 ml/kg and the other 20 ml/kg, within the first 12 hours after admission to PICU, in addition to continuous intravenous isotonic saline for hydration, and a 5 mg intravenous furosemide dose which resulted in a good diuresis response. Urine output (UOP) was not monitored accurately before the TVL was discovered but the patient was having normal wet diapers. Close monitoring of UOP was started after the first TVL. UOP was 2.0 ml/kg/h after the first isotonic saline bolus preceding any further treatment. Furosemide IV 5 mg was then given (0.83 mg/kg) followed by UOP 4.2 ml/kg/h during the first six hours, furosemide infusion (FI) was then started almost 11 hours later when UOP was 3.5 ml/kg/h. FI starting dose 0.07 mg/kg/h and fluid balances were monitored to avoid dehydration and overhydration. The dose range of FI was between 0.05-0.07 mg/kg/h for the first two days during which UOP ranged between 4.8-6.2 ml/kg/h. In the following two days, FI was decreased to 0.04 then 0.02 mg/kg/h, and then stopped on day 5 of furosemide therapy. UOP the following day after stopping was FI 2.6 ml/kg/h. The calculated estimated insensible water losses were ~ 92 ml/day. Fluid balances during FI days ranged between negative 52 ml to positive 179 ml. The patient was kept on IV fluid and was allowed for adlib breastfeeding. IV fluid was decreased gradually until it was stopped a day after FI discontinuation. The patient remained well hydrated during FI therapy and maintained normal hemodynamic status. Serum creatinine level peak was at 134 µmol/L on day 5 after starting vancomycin therapy, then it gradually decreased to 45 µmol/L and vancomycin level decreased to 3.2 μg/mL on day 11. Since audiology service was unavailable, the hearing was to be assessed in an outpatient clinic to evaluate for ototoxicity but the patient was a no-show for the appointment. No proof of medication preparation or administration errors was identified. After discharge, the patient was lost to follow-up.

## Discussion

Our literature review of pediatric patients with TVL identified 23 cases from 1992 to 2021 [8-27]. Table 1 presents a summary of the collected data.

**Table 1:** Literature review of pediatric patients with vancomycin intoxication AKI, acute kidney injury; UNK, unknown; M, male; F, female; ESKD, end stage kidney disease; CKD, chronic kidney disease; NA, not applicable; NL, normal; AB, abnormal; O, overdose; T, therapeutic; IHD, intermittent hemodialysis; CHARC HP, charcoal hemoperfusion; CVVHDF, continuous veno-venous hemodiafiltration; CVVH, continuous veno-venous hemofiltration; IHDF, intermittent hemodiafiltration; EBT, exchange blood transfusion; NGT, nasogastric tube.

Article	Age (months)	Sex	Body Weight in kg	Pre-existing Abnormal Baseline Serum Creatinine and/or Abnormal Baseline Kidney Structure	AKI Risk Factor other than Vancomycin	Vancomycin Dose	Peak Vancomycin Level (µg/mL)	AKI During Vancomycin Toxicity	Oligo-anuria	Intervention	Kidney Function Short Term Outcome	Irreversible Hearing Loss
Present Case Report	4	F	6.1	NO	NO	T	>400	YES	NO	FUROSEMIDE/HYDRATION	NL	UNK
Panzarino et al. [8]	14	F	8	YES (CKD)	YES	O	337.6	YES	UNK	IHD/CHARC HP	AB	NO
Bunchman et al. [9]	UNK	F	22	UNK	YES	UNK	345	YES	YES	IHD	UNK	UNK
Bunchman et al. [9]	UNK	M	5.6	YES (CKD)	YES	UNK	313	UNK	NO	IHD/CHARC HP	UNK	UNK
Goebel et al. [10]	0.20	M	3.73	YES (ESKD)	NA	O	240	NA	NA	CVVHDF	NA	UNK
Akil et al. [11]	204	F	38	YES (ESKD)	NA	O	101	NA	NA	IHDF	NA	UNK
Wu et al. [12]	156	M	UNK	YES (AKI)	YES	T	85.6	YES	UNK	NONE	NL	UNK
Soylu et al. [13]	26	F	10	YES (AKI)	YES	O	146	YES	YES	IHD (conventional)	NL	NO
Wicklow et al. [14]	96	M	UNK	NO	NO	T	45.8	YES	YES	IHD	NL	UNK
Burkhart et al. [15]	1.53	M	UNK	NO	YES	O	427	YES	YES	EBT/NGT CHARCOAL	NL	NO
Miner et al. [16]	1.76	F	UNK	UNK	YES	T	305	UNK	NO	NONE	NL	NO
Miner et al. [16]	0.3	F	UNK	NO	YES	T	368	YES	NO	NONE	NL	NO
Tissing et al. [17]	1	F	UNK	NO	YES	T	63.3	YES	YES	NONE	NL	NO
Muller et al. [18]	0.2	M	2.39	NO	YES	O	34.5	UNK	UNK	NONE	NL	NO
Muller et al. [18]	0.2	F	1.985	NO	YES	O	32	UNK	UNK	NONE	NL	NO
Balen et al. [19]	6	F	5.34	NO	YES	O	154	YES	UNK	NONE	NL	NO
Lemaire et al. [20]	2.4	UNK	3.3	NO	YES	O	222	YES	NO	IHD	NL	NO
Stidham et al. [21]	84	F	UNK	NO	YES	T	213	YES	YES	IHD/SALINE/ALBUMIN/FUROSEMIDE	NL	NO
Unal et al. [22]	0.53	F	1.38	NO	YES	O	84	NO	NO	EBT	NL	NO
Lv M et al. [23]	55	M	15.5	NO	YES	T	86	YES	YES	CVVHDF	NL	UNK
Shah et al. [24]	168	F	39	NO	YES	T	250	YES	YES	CVVH	NL	UNK
Ulinski et al. [25]	108	F	17	UNK	YES	O	420	YES	NO	IHD	NL	UNK
Ibach et al. [26]	96	F	29.9	NO	YES	T	37	YES	YES	FUROSEMIDE	NL	UNK
Pokorná et al. [27]	0.1	UNK	3.5	NO	YES	T	47	YES	YES	FUROSEMIDE	UNK	UNK

Four patients had pre-existing chronic kidney disease (CKD) or AKI [8,9,12,13], two had end-stage kidney disease (ESKD) maintained on kidney replacement therapy (KRT) [10,11], and three had unknown data [9,16,25].

After excluding two patients with ESKD, 95.2% had one or more risk factors for AKI other than vancomycin which included admission to the intensive care unit (ICU), birth at <32 weeks of gestation, hypotension, sepsis, nephrotoxic medications, pre-existing AKI or CKD, extracorporeal membrane oxygenation, and liver transplantation. Sixteen patients (73.9%) developed AKI [8,9,12,17,19-21,23-27] based on the definition by the Kidney Disease Improving Global Outcomes (KDIGO) 2012. One patient had almost unchanged serum creatinine [22]. 

TVL >100 μg/mL was reported in 14 cases. Ten patients had medication overdose, one of which had inadvertently received an undiluted dose [16]. Only two had TVL >400 µg/mL [15,25]. 

KRT can help increase vancomycin clearance and hemodialysis assists with relatively better clearance compared to peritoneal dialysis which provides slow clearance due to the relatively high molecular weight of the drug (∼1450 Da) [1,28]. Twelve patients received KRT in the form of intermittent hemodialysis (IHD) in nine patients [8,9,11,13,14,20,21,25] or continuous KRT in three patients [10,23,24], versus seven patients who received watchful management [12,16-19]. The intended meaning of watchful management is that no intervention was done for TVL and the patient continued to be monitored. Median TVL in patients who received KRT was 231 µg/mL (IQR 123.5-325.3) compared to the median TVL of 85.6 µg/mL (IQR 34.5-305) in patients who received watchful management. Patients who received KRT included two patients with pre-existing CKD [8,9], two with ESKD; one received hemodiafiltration [11] and the other received continuous KRT [10], and two with unknown kidney function or structure status [9,25]. The patients who received watchful management had normal baseline kidney function and/or structure except one patient [12] and one had unknown kidney function and structure status [16]. All patients who received KRT, after excluding patients with ESKD [10,11], had developed AKI [8,9,13,14,20,21,23-25] except one in whom the status of kidney function was unknown [9]. Four of the seven patients who received watchful management had developed AKI [12,16,17,19] and kidney function data during intoxication was unavailable for the remaining three patients [16,18]. Of the nine patients who received IHD, high flux dialyzer was used in seven, a conventional membrane dialyzer was used in one [13], and an unclear membrane type in one [8]. In patients who received IHD, most of them needed 2-3 sessions of treatment; vancomycin levels dropped 70%-90% from pre-intervention to sub-therapeutic ranges after completing treatment. A rebound increase in the vancomycin levels was observed between the IHD sessions. IHD using the conventional membrane reduced TVL from 131 μg/mL to <20 μg/mL [13]. Continuous KRT decreased vancomycin level by 70%-87% after 22-41 h of treatment [10,23,24]. In patients who received KRT, the short-term kidney function outcome improved in nine patients [14,20,21,23-25], was unknown in two other patients [9], and one patient with CKD became dialysis dependent [8]. On the other hand, all patients who received watchful management had normal serum creatinine in the short term [12,16-19].

Two babies underwent exchange blood transfusion (EBT) [15,22]. One had TVL with AKI and received in addition charcoal via a nasogastric tube [15]. The measured TVL immediately before EBT and 30 minutes after EBT were the same. Afterwards, the vancomycin level gradually dropped to < 20 μg/mL over the following three days while AKI is resolving [15]. Subsequently, serum creatinine was restored to normal levels [15]. The other patient had TVL with no AKI [22]. TVL immediately after EBT decreased by 79.7% and then to <1 μg/mL over the following 36 hours. Therefore, no conclusion can be drawn regarding the use of EBT or charcoal via nasogastric tube for clearance of TVL.

Two patients were treated with furosemide without dialysis [26,27]. One received furosemide infusion for several days, with serum creatinine being restored to normal [26]; the other had TVL of 47 μg/mL on day 6 with oliguric AKI on day 5. She was managed with furosemide. Vancomycin levels decreased to 30.6 μg/mL and serum creatinine level was 114 μmol/L on day 7, but no further data were available.

Watchful management was followed for seven patients, of whom six were between six days and six months of age [16-19] and one was older [12]. Four had associated nephrotoxicity (TVL 63-368 μg/mL) [12,16,17,19] and kidney function data during intoxication (TVL 32-305 μg/mL) was unavailable for three [16,18]. All patients had normal serum creatinine in the short term.

Our case had severe TVL and developed AKI stage 3 based on KDIGO AKI staging and had no other risk factors for AKI. The patient was started on furosemide therapy and intravenous fluid hydration. The short-term kidney function outcome was reassuring which is the same outcome in 17 (80.9%) of the reported 23 cases, after excluding patients with ESKD, regardless of whether intervention occurred and regardless of the severity of TVL. Only one patient with pre-existing CKD became dialysis-dependent [8]. The combination of vancomycin and furosemide therapy is thought to increase the risk of AKI [29]. We believe that such increased risk could be related to intravascular volume depletion caused by diuresis during concomitant vancomycin treatment. The use of furosemide in our patient was done while the patient’s hydration status was maintained to avoid intravascular depletion by using intravenous fluid in addition to allowing adlib breastfeeding. 

Acute tubular necrosis is the most common histopathological finding in the kidneys of patients with vancomycin toxicity. However, acute tubulointerstitial nephritis (AIN) has been described [30]. Because skin rash is seen in both vancomycin reaction and AIN, assessment for kidney function should be considered in vancomycin-treated patients if skin rash develops. Direct toxicity to the kidney caused by vancomycin has been questioned by experts in nephrology [31]. However, in our reported case, the patient did not have any other AKI risk factors apart from TVL. Hence, our case could support the argument of vancomycin direct kidney injury.

Twelve out of 23 cases underwent formal hearing assessment. Eleven had normal hearing [13,15-22], except for one patient who had transient impairment which resolved in a follow-up repeat assessment [8]. A retrospective case series showed similar results [32]. The remaining eleven cases did not report hearing assessment.

This case report of a very rare event is limited by the lack of data on long-term outcomes. Such cases may be underreported-hence, more cases are needed to help standardize how to approach such patients and to help in predicting long-term outcomes.

## Conclusions

In conclusion, TVL >400 µg/mL is rare in the pediatric literature. Children with TVL were managed differently and KRT was the most common intervention with encouraging short-term kidney function outcomes. Moreover, cases that received watchful management had favourable short-term outcomes, despite TVL >100 µg/mL in a few. Long-term kidney function outcome data were lacking. No irreversible hearing loss was found with TVL. Reporting of TVL in pediatric patients is recommended to help devise a protocol for the management of such cases and for predicting outcomes.
